# Enhancing wheat tolerance to salinity using nanomaterials, proline, and biochar-inoculated with *Bacillussubtilis*

**DOI:** 10.1016/j.heliyon.2024.e37160

**Published:** 2024-08-30

**Authors:** Muhammad Ayman, Mohamed A. Fahmy, Ahmed S.M. Elnahal, Haifa E. Alfassam, Hassan A. Rudayni, Ahmed A. Allam, Eman M. Farahat

**Affiliations:** a Department of Water and Soil Sciences, Faculty of Technology and Development, Zagazig University, Zagazig 44519, Egypt; b Department of Agricultural Microbiology, Faculty of Agriculture, Zagazig University, Zagazig 44519, Egypt; c Department of Plant Pathology, Faculty of Agriculture, Zagazig University, Zagazig 44519, Egypt; d Department of Biology, College of Science, Princess Nourah bint Abdulrahman University, P.O. BOX 84428, Riyadh, 11671, Saudi Arabia; e Department of Biology, College of Science, Imam Mohammad Ibn Saud Islamic University, Riyadh 11623, Saudi Arabia; f Department of Zoology, Faculty of Science, Beni-suef University, Beni-suef 65211 Egypt; g Minia Higher Technology Institute for Applied Health Sciences, Minia, Egypt

**Keywords:** Wheat plants, Salt stress, Nanoparticles, Plant growth promoting bacteria (PGPB), Biochar, Proline

## Abstract

Salinity negatively impacts crop production by affecting physiological and biochemical processes in plants. This study investigates the effectiveness of Nano-ZnO (NZn), proline (PA), Nano-TiO_2_ (NTi), Nano-SiO_2_ (NSi)), and biochar inoculated with *Bacillus subtilis* (OSBS) in enhancing wheat tolerance to salinity stress. Pot experiments were conducted under saline conditions with varying rates of biochar and foliar applications. Results indicated that 2 % OSBS with NZn and NSi significantly improved wheat growth, leaf area, and nutrient level, reducing the negative impacts of salinity.

## 1 Introduction

Salinity adversely affects crop yields, exacerbating agricultural challenges globally. This, together with global climate change and population growth, poses challenges to food security [1]. According to Mansoora et al. [2], 20 % of the entire irrigated field experienced deterioration, resulting in a one-third decrease in food output. The main causes of a 20%–50 % decrease in agricultural productivity worldwide are the impact of salinity and water shortages, which result in the redirection of photosynthetic energy from crop production to the plant's defense mechanisms against stress [3]. Of the entire 230 million hectares of irrigated land, 45 million hectares have become saline soil due to salt accumulation. Due to a lack of high-quality water, it is necessary to irrigate soils with brackish water [4]. Furthermore, salinity stress leads to osmotic stress, ion toxicity, and nutritional inequality, according to Ref. [3]. The presence of high levels of salt limits the growth and productivity of plants by causing changes in their metabolic pathways [5]. In addition, salt stress induces the generation of reactive oxygen species (ROS), and disrupts antioxidant defense mechanisms, photosynthetic activities, and hormone balance [6]. Moreover, it primarily affects other crucial growth-related processes such as nutrient availability, water relations, photosynthetic pigments, seed germination, photosynthetic mechanisms, and productivity [7]. Hence, for plants to survive in a saline environment, they must possess extremely efficient protective mechanisms to mitigate the harmful consequences caused by the formation of toxic metabolites generated by salt [2]. Many recent studies have provided applications for increasing the tolerance of plants to salt stress. In addition, these techniques enhance plants' tolerance against biotic and/or abiotic stress, especially salt stress, such as selection, breeding, genetic engineering, treating plants with supported materials under salt stress, biological techniques, etc. Recently, other techniques were applied, such as the application of biochar, plant growth-promoting bacteria (PGPB), proline, and some nanomaterials. Several studies indicated that biochar improves the physical, chemical, and biological characteristics of soil and holds nutrients side by side, serving as a tactic to sequester carbon in the soil and thus contributing to reducing greenhouse gases [[8], [9], [10]]. Biochar has a role in mitigating abiotic stresses such as drought, salinity, ion toxicity, high temperatures, etc. Recent studies indicated that biochar has an effective role in alleviating salt stress on wheat, maize, quinoa, and Alfalfa plants in salt-affected soil because it has a high surface area, high pores, many functional groups, adsorption of cations, and some heavy metals and Na [[8], [9], [10], [11], [12], [13]].

On the other hand, the PGPB has an effective role in promoting plant growth under salt stress. Consequently, the application of PGPB is attracting increasing concerns from researchers [[14], [15], [16], [17]]. In addition, several studies reported the benefits of PGPB under salt stress, such as *Bacillus, Azospirillum, Pseudomonas, Halomones, Azotobacter, Enterobacter,* and *Nitrosamines*, etc. [18,19]. Research has been conducted on the effectiveness of safeguarding crops against stressors, including drought, salinization, and heavy metal toxicity [20,21]. In addition, there are many recent studies in this context, such as the application of bacteria as PGPB, biofungicides, and bioinsecticides. This is mostly because of the comprehension of how bacteria inhibit the growth of microorganisms that harm plants [22], provide nutrients to plants [23], regulate plant hormones [24], and eliminate hazardous substances from the soil [25]. In addition, applying proline to the leaves is a commonly used treatment that has been found to have a significant impact on reducing the negative effects of salt stress on plants [26]. It is a widely recognized osmolyte that is produced in reaction to high salt levels. It is stored in plants in significant amounts and has a role in maintaining the equilibrium of water within cells, stabilizing proteins, removing harmful free radicals, and controlling gene expression. Proline functions as a signalling molecule that controls plant growth and development in response to stressful conditions [[27], [28], [29]]. Another treatment is the application of some nanomaterials. Nanomaterials are a dominant agricultural technology because of their ability to improve yield quantity and quality, decrease the usage of traditional pesticides and fertilizers by utilizing nano-size alternatives, and boost agricultural product yield [30]. Nanomaterials, which are particles that are a billionth of a meter in size, possess a significant surface area and exhibit heightened reactivity in several biological processes. Additionally, these materials are environmentally beneficial [31]. Presently, metal oxide nanoparticles like SiO_2_, TiO_2_, FeO, CeO_2_, Al_2_O_3_, and ZnO are being applied for several purposes such as fertilization, enhancing plant growth under stress conditions (biotic and abiotic stresses) such as salt stress. Their impact on disease management, toxicity, and improved grain production under unfavourable environmental conditions is currently being studied [[32], [33], [34]]. Certain nanomaterials serve as vital nutrients for plants, including calcium, iron, and zinc. Additionally, additional nanomaterials like silicon, titanium, and selenium are beneficial in improving the plant's capacity to withstand salt stress.

Additionally, wheat is the most important crop, experiencing a significant decrease in yield worldwide owing to saline stress [1]. Plants respond to salinity stress by closing stomata, inhibiting shoot growth, reducing the number of tillers, altering reproductive development, and lowering carbohydrate production, thus drastically affecting crop yield [35]. The deposition of soluble salts in the rooting medium of plants leads to hindered growth, reduced germination of kernels, impaired uptake of nutrients, compromised photosynthesis, damage to cell membranes, and the accumulation of toxins due to osmotic stress, imbalances in nutrition and hormones, ion toxicity, and oxidative damage [3]. Therefore, this study aimed to investigate the effect of OSBS application and foliar application of nano-Zn (NZn), proline (PA), nano-Ti (NTi), and nano-Si (NSi) in enhancing wheat tolerance to salt stress.

## 2 Materials and methods

### 2.1 Salt tolerance assay

The tested bacteria (*B. subtilis* subsp. *subtilis* AZFS3 (LC599401.1)), was already isolated and identified based on 16S rRNA gene sequencing, according to Fahmy et al. [36]. The mineral salt medium (MSM) consisted of (mg L^−1^): K_2_HPO_4_, 500; KH_2_PO_4_, 250; NaCl, 500; (NH_4_)_2_SO_4_, 230; CaCl_2_.2H_2_O, 7.5; MgSO_4_.7H_2_O, 100; MnSO_4_.7H_2_O, 100; FeCl_3_, 1 mg; Distilled water: 1000 mL at pH = 7.0., as described by Atlas et al. [37]**,** was used for the tolerance of NaCl by *B. subtilis* subsp. *subtilis* AZFS3. In brief*, B. subtilis* subsp. *subtilis* AZFS3 strains from stock cultures were aerobically subcultured in nutrient broth for bacteria at 30 °C for 18 h. An initial inoculum of approximately 10^7^ CFU mL^−1^ of the target isolate was incorporated into a nutrient broth tube (10 mL) and supplemented with variable concentrations of NaCl (0, 1, 2, 3, 5, 6, 7, 8, 9, and 10 %) in the tubes 0, control means that no salt is added to the MS broth during its preparation, which was incubated for 24 h at 30 °C, pH 7.0. The bacterial growth of the tested cultures was determined spectrophotometrically (UV-2101/3101 PC; Shimadzu Corporation, Analytical Instruments Division, Kyoto, Japan), which was used to measure the final optical density (OD) at a wavelength of 600 nm. This technique was employed according to Refs. [38,39].

### 2.2 Experimental design and treatments

Pot experiments were conducted to evaluate the effect of olive stone biochar inoculated with plant growth-promoting bacteria (*B. subtilis* subsp. *subtilis* AZFS3 (LC599401.1)) to soil and foliar application of distilled water (DW), NZn, PA, NTi, and NSi on wheat plants grown under salinity stress. The study included two factors in a split-plot design (SPD), the main factor was four rates of OSBS (0, 1, 2, and 5 %). The sub-factor included five treatments (DW, 50 mg NZn L^−1^, 50 mg PA L^−1^, 50 mg NTi L^−1^, and 80 mg NSi L^−1^). Twenty treatments were duplicated three times (4 (OSBS treatments) × 5 (foliar application treatments) × 3 replicates = 60 plots). The wheat seed cultivar named Giza 91 (*Triticum aestivum*) was procured from the Agriculture Research Centre (ARC) in Cairo, Egypt, and was cultivated in a pot experiment at the Faculty of Technology and Development greenhouse farm, Zagazig City, Egypt (location: latitude 30°35′23.7″ N, longitude 31°28′53.2″ E, on November 11, 2023–2024 (winter season).

Four rates of OSBS were inoculated with AZFS3 and mixed up thoroughly by the applied biochar at rates of 1 mL bacterial inoculum (1 mL of inoculum equivalent to 10^7^ CFU mL^−1^ from the tested bacterial strains) to 2-g biochar, respectively. All pots were taken, and each pot was filled with 6 kg of soil. The applied biochar has already been characterized in a previous paper [10]. Each pot was sown with seven seeds in uniform depth and space. All pots were irrigated with saline water (EC 2.67 dS m^−1^) 0.75L/pot every week. In addition, pots were sprayed one month after sowing with varying nanomaterials (DW; 50 mg NZn L^−1^; 50 mg NTi L^−1^; 80 mg NSi L^−1^) or proline (50 mg PA L^−1^) seven times up until the harvest stage. Plants were sampled one month after foliar application of nanomaterials or proline and at maturity to study various parameters.

### 2.3 Wheat growth parameters

Several wheat growth parameters were estimated such as stem length, root length, leaf area, leaf space guide, specific root length, fresh biomass and dry biomass yield.

Leaf area was calculated according to Miralles & Slafer [40]:

LeafArea(LA)=LW0.835;

where, L and W represent length, and width, respectively.

Leaf space guide was estimated according to Coombs et al. [41],

$$Leaf\;Space\;Guide\;\left(LSG\right)=\frac{Area\;of\;the\;plant^{\prime }s\;leaf\;surface\;\left({cm}^{2}\right)}{Area\;occupied\;by\;the\;plant\;\left({cm}^{2}\right)\;}$$

Specific root length (SRL) can be estimated according to Fageria [42]:

$$\left(SRL\right)=\frac{Root\;lenght\;\left(cm\right)}{Root\;dry\;weight\;\left(g\right)\;}$$

### 2.4 Soil, water, biochar, and plant analyses

Particle size distribution was determined using a soil hydrometer protocol. Bulk density (BD) and particle densities (PD) were measured using the core method and pycnometer, respectively. The soil reaction (pH) of the soil paste was determined using a pH-meter. Organic matter (OM) was assessed using a wet oxidation method (H_2_CrO_7_). The calcium and magnesium concentrations of soil and water samples (Ca^2+^+Mg^2+^) were titrated with EDTANa_2_. Extractable KCl-N was obtained by Kjeldahl methods. The electrical conductivity (EC) of the soil paste extracts was also determined using an EC meter. The cationic exchange capacity (CEC) was determined using 1M NaOAc. The extract of P was determined using a colourimetric method. Potassium (K) was measured using a flame photometer, whereas the extractable K from the soil and OSB samples were extracted using 1M NH4OAc. Analytical methods were performed as described by Estefan et al. [43]. Some physical and chemical properties of the soil, water, and biochar are presented in Table 1.

**Table 1:** Some physical and chemical analysis of tested soil, applied biochar, and used water.

Property	Unit	Soil	Biochar	Water
Physical properties				
Bulk density	g cm^−3^	1.56	–	–
Particle density	g cm^−3^	2.64	–	–
Particle size distribution				
Sand	g hg^−1^	45.90	–	–
Silt	g hg^−1^	31.30	–	–
Clay	g hg^−1^	22.80	–	–
Texture	class		–	–
Water-holding capacity	g hg^−1^	30.24	–	–
**Chemical properties**				
pH^a^	–	8.32	8.25	7.82
Electrical conductivity (EC**)	dS m^−1^	9.75	1.02	2.67
Cation exchange capacity (CEC)	cmole (+) Kg^−1^	18.27	83.34	–
Organic matter	g hg^−1^	1.09	92.12	–
CaCO_3_	g Kg^−1^	8.16	–	–
Extractable -N (KCl-N)	mg Kg^−1^	14.27	34.12	–
Olsen-P (NaHCO_3_-P)	mg Kg^−1^	21.34	163.11	–
Extractable -K (NaOAc-K)	mg Kg^−1^	186.12	8.12	–
Extractable -Si (CaCl_2_-Si)	mg Kg^−1^	27.64	–	–

a pH of soil and biochar was determined in a soil paste, and a 1:5 biochar suspension, respectively, ** EC of soil and biochar was determined in a soil paste, and a 1:5 biochar extracts, respectively.

The biochar sample and wheat samples were oven-dried, ground, and wet-digested using a mixture of H_2_SO_4_ and H_2_O_2_ at 420 °C for chemical analyses [44]. Total-N, P, and K levels were determined using the Kjeldahl, colourimetric, and flame-photometer techniques, respectively, according to Estefan et al. [43]. The EC of biochar was measured using 1:5 extract, whereas pH was measured using 1:5 suspension, as described by Pandian et al. [45]. The available N, P, and K of OSB were extracted with KCl, NaHCO_3_, and NH_4_OAc, respectively, as described by Estefan et al. [43]. The specific surface area of BET, EDAX, scanning electron microscope (SEM), and Fourier transform infrared (FTIR) of OSB was already characterized in a previous paper [10]. In addition, wheat samples were prepared for various parameters such as pigments (chlorophyll *a*, *b*, total, and carotenoids), non-enzymatic antioxidants (ascorbic acid), and organic osmolytes (free proline and total soluble sugars) using a specific preparation and measurement method applied by Lalarukh et al. [1].

### 2.5 Statistical analysis

For statistical evaluation, analysis of variance (ANOVA) was performed using a statistical package for the social sciences package (SPSS, V. 26). Standard errors (SE) of replicates and treatments were calculated, as well as the least significant difference (LSD) was calculated at a significant level of 5 % to evaluate differences between averages of treatments. All charts were made using the origin lab package (V.9).

## 3 Results

### 3.1 Salt tolerance resting of Bacillus subtilis subsp. subtilis (AZFS3)

**Fig. 1:**
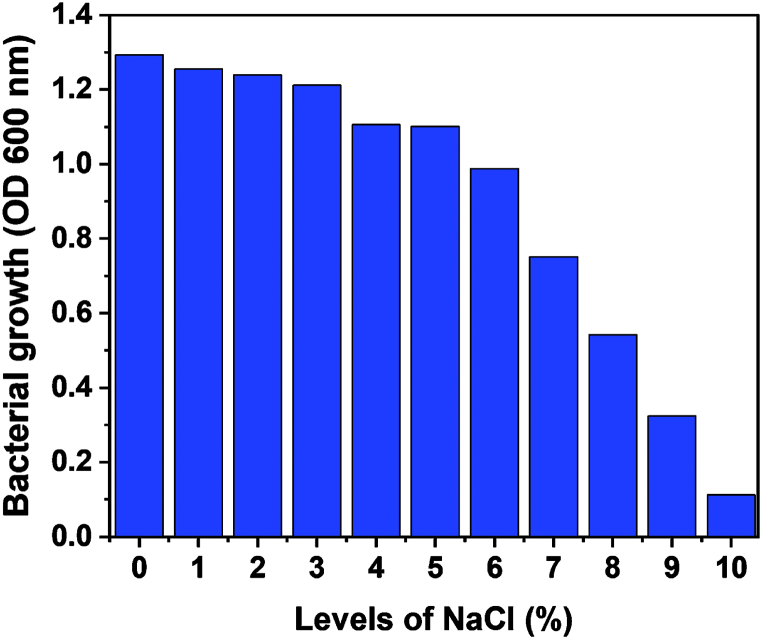
Bacterial growth (O.D. 600 nm) of the tested *Bacillus subtilis* subsp. *subtilis* AZFS3 strain in MSM medium supplemented with NaCl at different salinity levels.

In this experiment, the ability of *B. subtilis* subsp. *subtilis* AZFS3 strains to grow at different NaCl concentrations (0, 1, 2, 3, 5, 6, 7, 8, 9, and 10 %) was determined at 30 °C, pH 7.0, and incubation for 24 h. The experimental results in Fig. 1 showed the bacterial strain was very tolerant to the NaCl concentrations, as indicated by the obvious increase in their growth rates. *B. subtilis* subsp. *subtilis* AZFS3 showed tolerance up to 9 % against NaCl. Bacterial growth was observed at 0–9 %, and then it started declining, as shown in Fig. 1. The findings that were obtained in this study are consistent with Gul et al. [46] results showed that most bacterial species grew under 5 % NaCl concentration, while less growth was observed at 8 % and none at 10 % NaCl.

### 3.2 Effect of OSBS application, some nanomaterials, and proline on growth parameters of wheat plants, some physiological, and chemical characteristics

#### 3.2.1 Wheat growth parameters

**Fig. 2:**
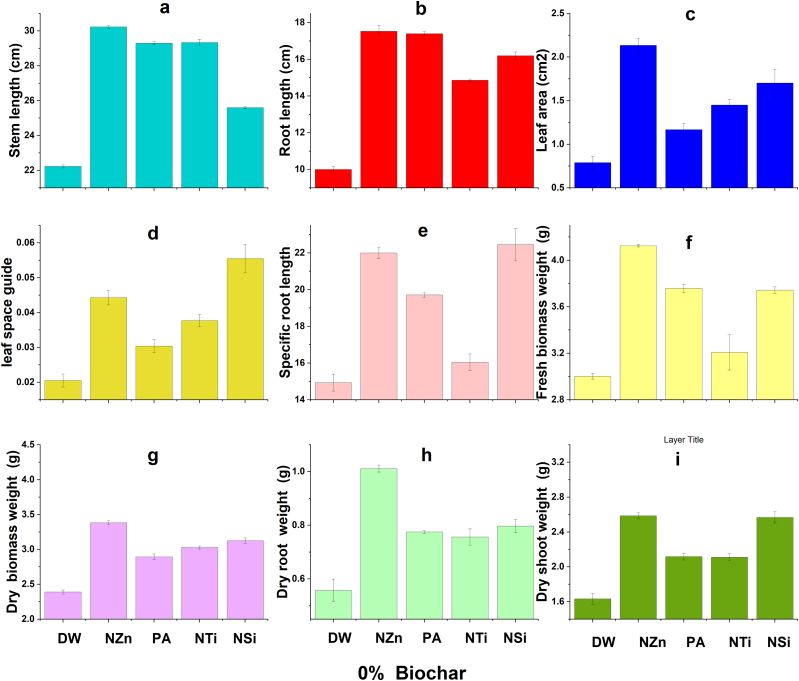
Effect of foliar application of distilled water (DW), nano-ZnO (NZn), proline acid (PA), nano-TiO_2_ (NTi), and nano-SiO_2_ (NSi) on stem length (a), root length (b), leaf area (c), leaf space guide (d), specific root length (e), fresh biomass weight (f), dry biomass weight (g), dry root weight (h), and dry shoots weight (g) at application rate of 0 % olive stone biochar inoculated with *B. subtilis* subsp. *subtilis* AZFS3.

**Fig. 3:**
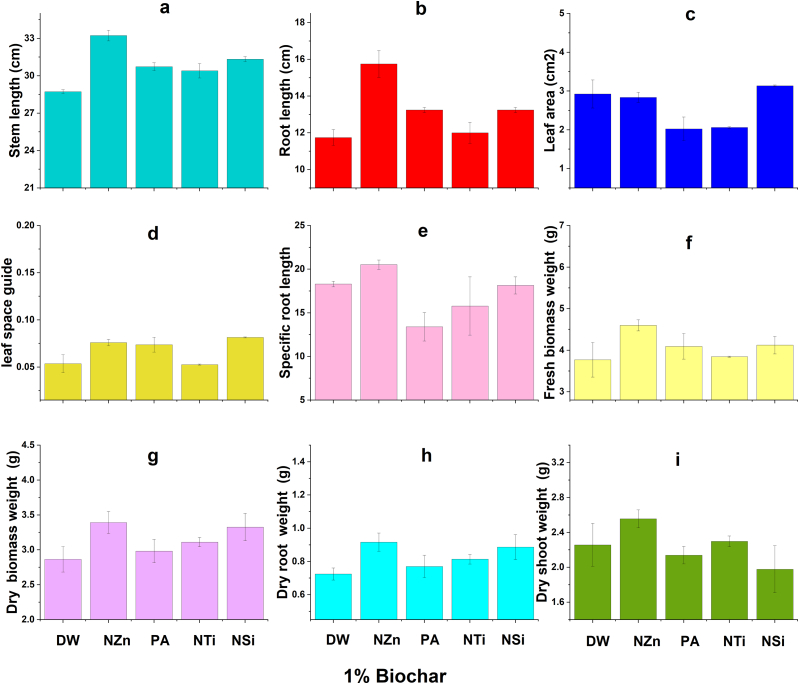
Effect of foliar application of distilled water (DW), nano-ZnO (NZn), proline acid (PA), nano-TiO_2_ (NTi), and nano-SiO_2_ (NSi) on stem length (a), root length (b), leaf area (c), leaf space guide (d), specific root length (e), fresh biomass weight (f), dry biomass weight (g), dry root weight (h), and dry shoots weight (g) at application rate of 1 % olive stone biochar inoculated with *B. subtilis* subsp. *subtilis* AZFS3.

**Fig. 4:**
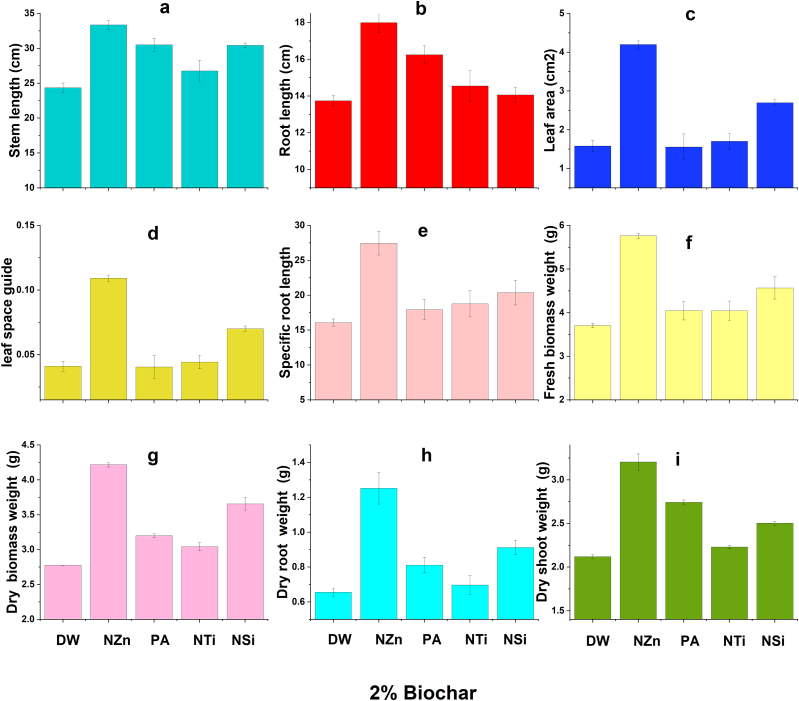
Effect of foliar application of distilled water (DW), nano-ZnO (NZn), proline acid (PA), nano-TiO_2_ (NTi), and nano-SiO_2_ (NSi) on stem length (a), root length (b), leaf area (c), leaf space guide (d), specific root length (e), fresh biomass weight (f), dry biomass weight (g), dry root weight (h), and dry shoots weight (g) at application rate of 2 % olive stone biochar inoculated with *B. subtilis* subsp. *subtilis* AZFS3.

**Fig. 5:**
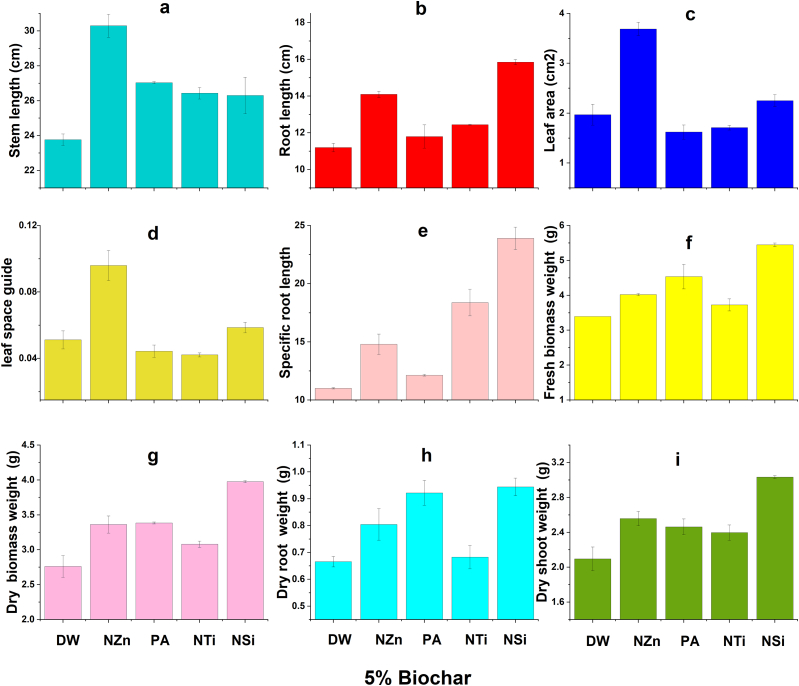
Effect of foliar application of distilled water (DW), nano-ZnO (NZn), proline acid (PA), nano-TiO_2_ (NTi), and nano-SiO_2_ (NSi) on stem length (a), root length (b), leaf area (c), leaf space guide (d), specific root length (e), fresh biomass weight (f), dry biomass weight (g), dry root weight (h), and dry shoots weight (g) at application rate of 5 % olive stone biochar inoculated with *B. subtilis* subsp. *subtilis* AZFS3.

The findings indicate that there were improvements in the growth characteristics of wheat under salt stress due to the application of OSBS and foliar application of nanomaterials and proline (Fig. 2, Fig. 3, Fig. 4, Fig. 5). Foliar application (FA) of nanoparticles and proline significantly increased most wheat growth parameters compared to the check treatment. The best treatments generally were the NZn treatment at a rate of 2%OSBS, then the NSi treatment at a rate of 5%OSBS (Fig. 2, Fig. 3, Fig. 4, Fig. 5i). The stem lengths varied between 22 and 33 cm, while the root lengths ranged from 10 to 18 cm, and the leaf space guides varied between 0.02 and 0.11 for the check treatment and NZn at the rate of 2%OSBS, respectively. Furthermore, there was an increase in both fresh and dry biomass values, with fresh biomass ranging from 3 to 5.7g, and dry biomass ranging from 2.4 to 4.2g for the same treatments. The increase in the biomass dry matter as a result of foliar application of NZn at the rate of 2%OSBS and NSi at the rate of 5%OSBS ranged from 76 to 66 %, respectively. The applied treatments resulted in a significant increase in stem length, root length, leaf space guides, fresh biomass, and dry biomass. These increases were an increase of 50 % in stem length, 80 % in root length, 432 % in leaf area index, 92 and 76 % in fresh and dry biomass, respectively. In contrast, NSi treatment at the rate of 5 % OSBS enhanced the same parameters by 18, 59, 185, 81, and 66 %, respectively, compared to the check treatment without OSBS or FA. Growth parameters of check treatment generally decreased in a noticeable decline when compared to the other treatments due to the salinity stress of soil and water.

#### 3.2.2 Level of chlorophyll, carotenoids, proline, and total soluble sugars

Results obtained in Table (2), the levels of pigments such as chlorophyll A (Ch. A), B (Ch. B), total chlorophylls (Ch.T), and carotenoids (Cart) of wheat tissues were affected by salt stress. The values of these parameters ranged from 1.59 to 8.56 mg g^−1^, 1.4–6.7 mg g^−1^, 2.2–9.8 mg g^−1^, and 0.04–2.5 mg g^−1^, respectively. The foliar application by DW generally recorded the highest values in pigments, while the NSi treatment recorded the lowest values in the levels of Ch. A and B and total. In addition, the highest values of carotenoid were at a rate of 1%OSBS and NSi treatment (0.97, and 1.23 mg g^−1^), while the foliar application of PA, and OSBS application rate of 2 % OSBS had the lowest values (0.20, and 0.48 mg g^−1^) (Table 3). The levels of PA (organic osmolytes) in plant tissues generally increased due to increasing rates of biochar application and spraying by NZn, while PA decreased with foliar application by PA and NTi (Table 3). Foliar application of NZn treatment and application of OSBS at a rate of 2 %, recorded the highest values in the proline level. The values of ascorbic acid (an antioxidant) decreased due to increasing OSBS rates and foliar application of nanomaterials and proline treatments, except at the rate of 2 %, whose values ranged from 0.16 to 1.17 μg g^−1^ (Table 2, Table 3). Total soluble sugars (TSS) (organic osmolytes) levels ranged from 0.12 to 0.65 μmol g^−1^ (Table 2), and it increased only at the rate of 2 % (Table 2). The spraying with NTi recorded the highest value, while the check treatment (DW) had the lowest value (Table 3).

**Table 2:** Effect of olive stone biochar inoculated with *Bacillus subtilis* subsp. *Subtilis AZFS3* (OSBS), and foliar application (FA) of some materials (distilled water (DW), nano-ZnO (NZn), proline acid (PA), nano-TiO_2_ (NTi), and nano-SiO_2_ (NSi), on the content of chlorophyll A (Ch. A), chlorophyll B (Ch. B), total chlorophyll (Ch. T), carotenoids (Cart.), proline acid (PA), ascorbic acid (AsA), and total soluble sugars (TSS) of wheat plants under salinity conditions.

OSBS rates (g hg^−1^)	(FA)	Ch. A (mg g^−1^ FB)			Ch. B (mg g^−1^ FB)			Ch. T (mg g^−1^ FB)			Cart. (mg g^−1^ FB)			PA (μg g^−1^DB)			AsA (μg g^−1^DB)			TSS (μmol g^−1^ DB)		
**0**	**DW**	8.85	±	0.63	4.73	±	0.45	7.93	±	0.39	0.26	±	0.25	13.10	±	0.17	1.15	±	0.02	0.12	±	0.01
	**NZn**	8.56	±	0.16	3.61	±	0.04	6.99	±	0.01	0.60	±	0.02	20.79	±	0.18	0.78	±	0.32	0.15	±	0.00
	**PA**	3.44	±	0.49	3.80	±	0.03	5.85	±	0.04	0.08	±	0.02	11.86	±	0.09	0.58	±	0.02	0.12	±	0.01
	**NTi**	7.90	±	0.69	4.57	±	0.09	5.81	±	0.02	0.87	±	0.05	9.28	±	0.18	1.02	±	0.07	0.65	±	0.01
	**NSi**	3.30	±	0.38	4.91	±	0.07	5.87	±	0.04	1.93	±	0.04	9.44	±	0.18	0.24	±	0.00	0.14	±	0.00
**1**	**DW**	6.66	±	0.20	1.76	±	0.48	6.56	±	0.07	2.53	±	0.29	14.73	±	0.38	0.89	±	0.14	0.13	±	0.01
	**NZn**	6.74	±	0.05	5.58	±	0.15	9.75	±	0.04	0.56	±	0.19	23.03	±	1.09	0.56	±	0.10	0.18	±	0.01
	**PA**	4.42	±	0.02	5.89	±	1.44	7.13	±	0.99	0.13	±	0.06	15.59	±	0.55	0.19	±	0.02	0.13	±	0.01
	**NTi**	3.16	±	0.11	3.34	±	0.15	7.41	±	0.26	1.13	±	0.18	11.75	±	0.88	0.72	±	0.07	0.13	±	0.01
	**NSi**	2.74	±	0.09	2.42	±	0.58	3.99	±	0.72	0.49	±	0.07	22.60	±	0.05	0.74	±	0.01	0.39	±	0.14
**2**	**DW**	4.38	±	0.03	6.75	±	0.41	6.33	±	0.38	0.46	±	0.21	33.70	±	0.98	0.70	±	2.22	0.23	±	0.02
	**NZn**	6.86	±	1.08	4.38	±	0.30	5.15	±	0.35	0.17	±	0.08	54.75	±	5.55	2.84	±	0.24	0.55	±	0.01
	**PA**	2.42	±	0.67	5.99	±	0.12	6.64	±	0.49	0.14	±	0.06	28.21	±	0.14	0.32	±	0.15	0.35	±	0.02
	**NTi**	4.16	±	0.42	2.48	±	0.76	4.54	±	0.90	0.29	±	0.14	21.89	±	1.18	0.16	±	0.04	0.36	±	0.01
	**NSi**	8.07	±	0.33	1.44	±	0.03	5.84	±	0.16	1.36	±	0.16	29.76	±	1.07	0.53	±	0.21	0.37	±	0.03
**5**	**DW**	5.62	±	1.32	2.24	±	0.63	9.82	±	1.30	0.83	±	0.14	23.16	±	0.38	0.34	±	0.04	0.17	±	0.00
	**NZn**	3.70	±	0.49	5.55	±	1.47	6.97	±	1.57	0.04	±	0.02	32.24	±	1.36	0.54	±	0.17	0.18	±	0.06
	**PA**	5.58	±	2.02	5.15	±	0.81	7.69	±	0.43	0.47	±	0.24	19.53	±	0.39	0.35	±	0.16	0.22	±	0.00
	**NTi**	4.98	±	1.38	3.61	±	0.54	6.00	±	0.31	0.47	±	0.26	11.01	±	2.99	0.22	±	0.03	0.16	±	0.04
	**NSi**	1.59	±	0.10	1.51	±	0.70	2.23	±	0.67	1.15	±	0.11	25.42	±	0.32	0.55	±	0.22	0.26	±	0.01

In this Table; FB, fresh biomass; DB, dry biomass; and all values are Mean ± standard error.

**Table 3:** Statistical analysis and interaction effect between olive stone biochar inoculated with *Bacillus subtilis* subsp. *Subtilis AZFS3* (OSBS), and foliar application (FA) of some materials (distilled water (DW), nano-ZnO (NZn), proline acid (PA), nano-TiO_2_ (NTi), and nano-SiO_2_ (NSi), on the content of chlorophyll A (Ch. A), chlorophyll B (Ch. B), total chlorophyll (Ch. T), carotenoids (Cart.), proline acid (PA), ascorbic acid (AsA), and total soluble sugars (TSS) of wheat plants under salinity conditions.

OSBS rates (g hg^−1^)	Ch. A (mg g^−1^ FB)			Ch. B (mg g^−1^ FB)			Ch. T (mg g^−1^ FB)			Cart. (mg g^−1^ FB)			PA (μg g^−1^DB)			AsA (μg g^−1^DB)			TSS (μmol g^−1^ DB)		
**0**	6.41a	±	1.25	4.32ns	±	0.26	6.49ns	±	0.42	0.75b	±	0.33	12.90d	±	2.10	0.75b	±	0.16	0.24b	±	0.10
**1**	4.75b	±	0.84	3.80ns	±	0.83	6.97ns	±	0.92	0.97a	±	0.42	17.54c	±	2.25	0.62c	±	0.12	0.19b	±	0.05
**2**	5.18b	±	1.01	4.21ns	±	1.01	5.70ns	±	0.38	0.48c	±	0.23	33.66a	±	5.60	0.91a	±	0.49	0.37a	±	0.05
**5**	4.29b	±	0.76	3.61ns	±	0.79	6.54ns	±	1.25	0.59BCE	±	0.19	22.27b	±	3.50	0.40d	±	0.06	0.20b	±	0.02
**FA**																					
**DW**	6.38a	±	0.95	3.87b	±	1.16	7.66a	±	0.80	1.02a	±	0.52	21.17b	±	4.72	1.18a	±	0.56	0.16c	±	0.02
**NZn**	6.46a	±	1.01	4.78b	±	0.48	7.22b	±	0.95	0.34c	±	0.14	32.70a	±	7.75	0.77b	±	0.17	0.26b	±	0.09
**PA**	3.96b	±	0.68	5.21a	±	0.51	6.83b	±	0.39	0.20c	±	0.09	18.80c	±	3.51	0.36d	±	0.08	0.21c	±	0.05
**NTi**	5.05b	±	1.02	3.50b	±	0.43	5.94b	±	0.59	0.69b	±	0.19	13.48d	±	2.85	0.53c	±	0.21	0.33a	±	0.12
**NSi**	3.92b	±	1.43	2.57b	±	0.81	4.48c	±	0.87	1.23a	±	0.30	21.81b	±	4.38	0.51e	±	0.10	0.29 ab	±	0.06
**Interaction**																					
**OSBS**	Sig.			NS			NS			Sig.			Sig.			Sig.			Sig.		
**FA**	Sig.			Sig.			Sig.			Sig.			Sig.			Sig.			Sig.		
**OSBS×FA**	Sig.			Sig.			Sig.			Sig.			Sig.			Sig.			Sig.		

In this Table: All values are Mean ± standard error.

#### 3.2.3 Levels of nitrogen, phosphorus, and potassium

The level of nitrogen (N), phosphorus (P), and potassium (K) in wheat shoots ranged from 0.69 to 1.10 g hg^−1^, 0.00–0.04 g hg^−1^, and 4.02–8.44 g hg^−1^, respectively (Table 4). In contrast, the levels of N, P, and K in wheat roots ranged from 0.35 to 0.49, 0.01–0.02, and 2–3 g hg^−1^, respectively (Table 4). In general, the levels of N, P, and K of wheat roots improved due to OSBS application, especially at the rate of 2 % with foliar application of NZn treatment. Foliar application with NZn recorded the highest absorption rates of N (roots, shoots), and K in the roots, while treatment with OSBS at the rate of 2 % recorded the highest absorption levels of N and P in the wheat roots and shoots. In addition, the OSBS application rate of 5 % recorded the highest rates of K absorption in the wheat roots and shoots (Table 5). Therefore, foliar application with NZn achieved the best growth of wheat under salinity conditions, and application of OSBS at the rate of 2 % achieved significant improvements in most growth parameters.

**Table 4:** Effect of olive stone biochar inoculated with *Bacillus subtilis* subsp. *Subtilis AZFS3* (OSBS), and foliar application (FA) of some materials (distilled water (DW), nano-ZnO (NZn), proline acid (PA), nano-TiO_2_ (NTi), and nano-SiO_2_ (NSi)), on the content of nitrogen (N), phosphorus (P), and potassium (K), in wheat shoots and roots.

OSBS rates (g hg^−1^)	(FA)	N (g hg^−1^)						P (g hg^−1^)						K (g hg^−1^)					
		Shoot			Root			Shoot			Root			Shoot			Root		
**0**	**DW**	0.76	±	0.05	0.38	±	0.01	0.041	±	0.0010	0.006	±	0.000	6.84	±	0.12	2.02	±	0.12
	**NZn**	0.83	±	0.05	0.42	±	0.02	0.021	±	0.0004	0.006	±	0.001	6.84	±	0.12	3.09	±	0.07
	**PA**	0.74	±	0.03	0.35	±	0.01	0.023	±	0.0003	0.006	±	0.001	7.10	±	0.07	2.55	±	0.07
	**NTi**	0.81	±	0.15	0.36	±	0.01	0.008	±	0.0009	0.007	±	0.003	5.63	±	0.12	2.42	±	0.12
	**NSi**	0.77	±	0.09	0.46	±	0.01	0.031	±	0.0016	0.010	±	0.002	6.63	±	0.00	2.95	±	0.07
**1**	**DW**	0.69	±	0.01	0.39	±	0.01	0.031	±	0.0066	0.008	±	0.001	5.73	±	0.29	2.35	±	0.07
	**NZn**	1.00	±	0.08	0.40	±	0.00	0.027	±	0.0006	0.016	±	0.001	6.73	±	0.64	2.95	±	0.07
	**PA**	0.89	±	0.09	0.39	±	0.01	0.026	±	0.0006	0.006	±	0.001	6.43	±	0.00	2.55	±	0.07
	**NTi**	0.84	±	0.07	0.37	±	0.00	0.014	±	0.0008	0.003	±	0.000	6.13	±	0.75	2.95	±	0.07
	**NSi**	0.85	±	0.06	0.37	±	0.02	0.020	±	0.0040	0.013	±	0.001	4.83	±	0.23	2.35	±	0.07
**2**	**DW**	0.70	±	0.03	0.35	±	0.01	0.024	±	0.0042	0.010	±	0.001	6.94	±	0.06	2.55	±	0.07
	**NZn**	1.02	±	0.11	0.49	±	0.01	0.019	±	0.0034	0.018	±	0.000	8.34	±	0.17	2.89	±	0.07
	**PA**	1.08	±	0.09	0.37	±	0.00	0.013	±	0.0014	0.016	±	0.001	8.44	±	0.46	2.62	±	0.12
	**NTi**	1.09	±	0.06	0.43	±	0.00	0.005	±	0.0001	0.014	±	0.001	4.02	±	0.23	2.69	±	0.07
	**NSi**	0.87	±	0.09	0.39	±	0.00	0.019	±	0.0005	0.018	±	0.000	6.63	±	0.12	2.95	±	0.07
**5**	**DW**	0.84	±	0.03	0.36	±	0.01	0.019	±	0.0018	0.005	±	0.000	8.34	±	0.87	2.28	±	0.07
	**NZn**	1.09	±	0.01	0.39	±	0.01	0.022	±	0.0020	0.014	±	0.001	7.44	±	0.70	2.89	±	0.07
	**PA**	0.79	±	0.00	0.37	±	0.00	0.015	±	0.0017	0.009	±	0.001	7.04	±	0.12	2.95	±	0.07
	**NTi**	1.10	±	0.00	0.40	±	0.00	0.003	±	0.0016	0.012	±	0.000	6.43	±	0.35	2.75	±	0.07
	**NSi**	0.75	±	0.01	0.44	±	0.01	0.019	±	0.0063	0.017	±	0.001	6.94	±	0.06	2.95	±	0.07

In this Table: All values are Mean ± standard error, g hg^−1^ is g per hectogram (100g).

**Table 5:** Statistical analysis and interaction effect between olive stone biochar inoculated with *Bacillus subtilis* subsp. *Subtilis AZFS3* (OSBS), and foliar application (FA) of some materials (distilled water (DW), nano-ZnO (NZn), proline acid (PA), nano-TiO_2_ (NTi), and nano-SiO_2_ (NSi)), on the content of nitrogen (N), phosphorus (P), and potassium (K), in wheat shoots and roots.

OSBS rates (g hg^−1^)	N (g hg^−1^)						P (g hg^−1^)						K (g hg^−1^)					
	Shoot			Root			Shoot			Root			Shoot			Root		
**0**	0.78b	±	0.02	0.39b	±	0.02	0.02a	±	0.01	0.01c	±	0.001	6.61b	±	0.26	2.61b	±	0.19
**1**	0.86 ab	±	0.05	0.38c	±	0.01	0.02a	±	0.00	0.01BCE	±	0.002	5.97c	±	0.32	2.63b	±	0.14
**2**	0.95a	±	0.08	0.41a	±	0.03	0.02b	±	0.00	0.02a	±	0.002	6.88 ab	±	0.80	2.74a	±	0.08
**5**	0.91 ab	±	0.08	0.39b	±	0.01	0.02b	±	0.00	0.01b	±	0.002	7.24a	±	0.33	2.77a	±	0.13
**FA**																		
**DW**	0.75c	±	0.03	0.37c	±	0.01	0.03a	±	0.00	0.01c	±	0.001	6.96a	±	0.53	2.30c	±	0.11
**NZn**	0.99a	±	0.05	0.43a	±	0.02	0.02a	±	0.00	0.01a	±	0.003	7.34a	±	0.37	2.95a	±	0.05
**PA**	0.88 ab	±	0.07	0.37c	±	0.01	0.02b	±	0.00	0.01b	±	0.002	7.25a	±	0.42	2.67b	±	0.10
**NTi**	0.96a	±	0.08	0.39b	±	0.02	0.01c	±	0.00	0.01b	±	0.002	5.56c	±	0.54	2.70b	±	0.11
**NSi**	0.81BCE	±	0.03	0.42a	±	0.02	0.02b	±	0.00	0.01a	±	0.002	6.26b	±	0.48	2.80b	±	0.15
**Interaction**																		
**OSBS**	Sig.			Sig			Sig.			Sig.			Sig.			Sig.		
**FA**	Sig.			Sig.			Sig.			Sig.			Sig.			Sig.		
**OSBS×FA**	Sig.			Sig.			Sig.			Sig.			Sig.			Sig.		

In this Table: All values are Mean ± standard error, g hg^−1^ is g per hectogram (100g).

## 4 Discussion

According to the results in the previous section (Fig. 1), it is clear to us that the AZFS3 strain is significantly tolerant of salinity even under high levels of salinity (up to 5%NaCl). In addition, this result agreed with result by Fahmy et al. [36], which confirmed that this strain tolerates high levels of salinity. They showed that most bacterial species grew under 5 % NaCl concentration, while less growth was observed at 8 % and none at 10 % NaCl. In addition, wheat growth parameters were affected by salt stress conditions, Lalarukh et al. agreed with us that increased salinity generally reduces most plant growth [1], and this can be attributed to the negative effect of salts such as ionic toxicity, increased formation of reactive oxygen species (ROS), and the effect on cell division, elongation, and organelles, which also causes widespread oxidative damage to cells and their organelles [43]. Therefore, wheat growth decreases under conditions of salt stress in soil or water. These results also agree with those of El-Bassiouny et al. [47], who recorded significant reductions in plant morphological parameters under stressful conditions such as salinity.

Generally, there is a significant increase in most wheat growth parameters like plant height, root length, leaf area, and biomass yield owing to foliar application. According to several studies, these foliar applications such as the foliar application of NZn [1,48,49], PA [29], NTi [50,51], and NSi [33,52], played considerable roles in the alleviation of salt-stressed wheat plants by regulating their tolerance mechanisms. Zinc is known to be one of the essential nutrients that plants need to complete their life cycle. It has many direct and indirect roles in the plant that play a role in enhancing plant growth against salt stress. Silicon also has positive roles in enhancing plant growth under biotic and abiotic stress conditions like salinity. Consequently, Zn and Si have nutritional roles in most plants, as well as these beneficial elements help plants tolerate salt stress in soil and/or irrigation water [33,48,52]. Foliar application of proline, zinc and titanium oxide also supports plant growth grown under abiotic stress like salinity [29,[49], [50], [51]]. In addition, biochar can adsorb cations such as Na^+^ ions and thus reduce the effect of cationic ions, due to its high porosity, surface area, cation exchange capacity, functional groups and water-holding capacity [[9], [10], [11], [12], [13]]. In contrast, salinity stress negatively affected in the check treatments compared to foliar-applied treatments. Because salinity obstructs many physiological [1,[53], [54]] and biochemical activities in plants [1,49], hence, this stress suppresses their growth [51]. The negative effect of salinity was also noticed on the roots and shoot of *Lupinus termis* [55], *Solanum tuberosum* [48], *Rosmarinus officinalis* [56] and *Vicia faba* [57]. Foliar application is an important way to enhance the growth of wheat plants under salinity stress conditions. In general, most wheat growth parameters improved due to the synergistic application of biochar with tested strain (Fig. 2a–i), especially at a rate of 2 %, while foliar application, especially with NZn and NSi. This can also be attributed to biochar has a porous structure and a large surface area, which allows it to absorb microorganisms and organic chemicals [58]. Biochar additionally shields them from harmful effects of some pathogens in soil [59]. Due to its high carbon level, which serves as a substrate, and the presence of vital nutrients, this substance supplies both energy and the necessary building blocks for the survival and growth of inoculants [60]. Furthermore, biochar alters the physicochemical characteristics of soils, which can potentially result in an augmentation of soil microbial biomass and enzymatic activity [61]. Biochar contains various mineral nutrients such as nitrogen, phosphorus, potassium, calcium, magnesium, zinc, etc., which vary depending on the type of feedstock and the temperature used during pyrolysis [62]. Thus, biochar can enhance the availability of some nutrients to the plant along with the bacterial activity of the strain used to promote wheat growth under these saline conditions. When added to soil, it undergoes progressive decomposition, releasing these nutrients into the soil solution [63]. This demonstrates that foliar application and synergistic application of biochar with *Bacillus* mitigated the harmful effects of salinity on the growth of wheat plants. Other studies have also indicated that biochar has many pores, a high surface area, and functional groups that preserve nutrients, water-holding capacity and adsorb of Na^+^ ions. Therefore, it has an important role in promoting the growth of wheat under saline conditions. In addition, biochar is considered a home for microbes and thus enhances biological diversity in the soil under salinity conditions.

For foliar application with nanomaterials and proline, there is a significant increase in wheat growth parameters like wheat height, root length, leaf area, and biomass yield owing to foliar application of NZn [1,48,49,57], proline [29], NTi [50,51], and NSi [33,52]. According to previous studies, this foliar application of applied materials played considerable roles in the alleviation of salt-stressed wheat plants by regulating their tolerance mechanisms. Our results were consistent with the results of studies conducted to determine the effect of foliar application of NZn [48], PA [29], NTi [50,51], and NSi [33,48,52]. These studies confirmed that the foliar application of these materials has a positive effect in increasing the ability of wheat to withstand stress. Some studies also indicated that the synergistic application of biochar with growth-promoting bacteria, especially *Bacillus*, has an important role in alleviating the harmful effects of salinity on wheat growth under saline conditions. In addition, the chlorophyll levels of wheat leaves (a, b, and total) improved with the enhanced application of biochar (2 %) and the foliar application of various materials, especially NSi. In contrast, the rate of 1 % and the application of silicon recorded the highest values in the cartonide level. The proline level also increased with the increase in the rates of OSBS. Our results are like the findings of Babaei et al. [64] who noticed significant reductions in chlorophyll level in plants. We can interpret this as foliar applications and the application of OSBS alleviate salt stress by enhancing wheat pigments, according to Refs. [9,10]. In addition, spraying plants with nanoparticles and PA significantly decreased some pigments in wheat plants. This finding was also recorded by Abdel Latef et al. [55] in lupin plants. It is worth mentioning that this result probably did not contradict the findings, which indicate that this foliar spray significantly improved plant growth parameters, especially with increasing its rate of application up to 80 mg L^−1^ as the rate of plant growth owing to this spray could be much higher than the rate of formation of these pigments in the plant. Moreover, these results are like Lalarukh et al. [1] who recorded significant decreases in the enzymatic antioxidant (catalase) in plants sprayed with NZn. Although these antioxidants are needed to increase plant tolerance to salinity [65], the decrease in the activities of antioxidant enzymes may indicate that this nano-product successfully lessened the implications of salinity stress in plants.

On the other hand, organic osmolytes (PA and TSS) increased significantly in the leaves of the studied wheat under salinity stress conditions and further increased with the foliar application of NZn, and NSi particles, at a rate of 2 % and vice versa. In contrast, TSS increased in all applied treatments at a rate of 2 % OSBS. This modulation (proline and total soluble sugar) could improve the osmotic protection of plant cells [47,64]. We can attribute the improvements, especially at the application rate of 2 % OSBS under salt stress conditions, to the fact that the biochar has a high absorption capacity and surface area for absorbing dissolved salts, especially cationic ions such as Na, according to Ayman et al. [8], Ayman and Fawzy [10]. In addition, recent studies have shown that plants inoculated with *Bacillus gypsum* improved their growth traits, and reduced the harmful effects of salinity stress, such as the accumulation of malondialdehyde and hydrogen peroxide [46]. Moreover, inoculation with biochar leads to increased absorption of essential nutrients in several plants such as wheat, rice, maize, cucumber, and tomato [46,[66], [67], [68], [69], [70]]. The results of our study are consistent with results of studies that confirmed the positive effects of biochar [46,[69], [70], [71], [72]]. The ascorbic acid levels, N, P, and K levels of leaves and roots were improved due to the application of OSBS, and foliar application of nanomaterials, and proline. In addition, recent studies have shown that plants inoculated with *Bacillus gypsum salum* improved their growth traits, and reduced the harmful effects of salinity stress, such as the accumulation of malondialdehyde and hydrogen peroxide [46]. Moreover, inoculation with biochar leads to increased absorption of essential nutrients in several plants such as wheat, rice, maize, cucumber, and tomato [46,[66], [67], [68], [69], [70]]. More comprehensively, it can be said that the best-applied treatments were a foliar spray of NZn and an application of OSBS at the rate of 2 %.

## 5 Conclusions

Foliar application of nanoparticles and proline effectively lessens the negative effects of salinity stress on wheat plants. Application of nano-Zn particles (50 mg L^−1^) with biochar inoculated with *B. subtilis* subsp. *subtilis* AZFS3 under salt stress conditions also enhanced the growth of either wheat plant in terms of root and shoot (fresh and dry) weights, shoot and root lengths, as well as plant leaf area index. Furthermore, nano-Zn and nano-Si particles foliar application led to statistical reductions in plant pigments (i.e., carotenoids, chlorophyll A, B, and their total levels) and non-enzymatic (ascorbic acid) antioxidants in plants. Yet, this foliar spray enhanced the formation of total soluble sugars and proline. Moreover, this addition of OSBS at a rate of 2 % significantly raised wheat growth parameters and NPK levels in wheat roots and shoots. The study concludes that foliar application of nano-Zn and Si, combined with 2 % biochar inoculated with *Bacillus subtilis*, enhances wheat growth under salinity stress.

## Data availability statement

All data are contained within the article.

## Supplementary data

There is no supplementary data.

## Funding

This work has received a fund from Princess Nourah bint Abdulrahman University Researchers Supporting Project number (PNURSP2024R400), Princess Nourah bint Abdulrahman University, Riyadh, Saudi Arabia.

## CRediT authorship contribution statement

**Muhammad Ayman:** Writing – review & editing, Writing – original draft, Supervision, Software, Resources, Project administration, Methodology, Investigation, Formal analysis, Data curation, Conceptualization. **Mohamed A. Fahmy:** Writing – review & editing, Validation, Software, Methodology, Formal analysis, Data curation, Conceptualization. **Ahmed S.M. Elnahal:** Writing – review & editing, Writing – original draft, Software, Methodology, Formal analysis, Data curation, Conceptualization. **Haifa E. Alfassam:** Visualization, Software, Funding acquisition. **Hassan A. Rudayni:** Visualization, Software, Funding acquisition. **Ahmed A. Allam:** Visualization, Software, Funding acquisition. **Eman M. Farahat:** Writing – review & editing, Writing – original draft, Visualization, Validation, Supervision, Software, Resources, Methodology, Investigation, Formal analysis, Data curation, Conceptualization.

## Declaration of competing interest

The authors declare that they have no known competing financial interests or personal relationships that could have appeared to influence the work reported in this paper.

## References

[1] Lalarukh I., Zahra N., Al Huqail A.A., Amjad S.F., Al-Dhumri S.A., Ghoneim A.M., Alshahri A.H., Almutari M.M., Alhusayni F.S., Al-Shammari W.B., Poczai P., Mansoora N., Ayman M., Abbas M.H.H., Abdelhafez A.A. (2022). Exogenously applied ZnO nanoparticles induced salt tolerance in potentially high yielding modern wheat (*Triticum aestivum* L.) cultivars. Environ. Technol. Innov..

[2] Mansoora N., Kausar S., Amjad S.F., Yaseen S., Shahid H., tul Kubra K., Alamri S.A.M., Alrumman S.A., Eid E.M., Mustafa G., Ali S.A., Danish S. (2021). Application of sewage sludge combined with thiourea improves the growth and yield attributes of wheat (*Triticum aestivum* L.) genotypes under arsenic-contaminated soil. PLoS One.

[3] Amjad S.F., Mansoora N., Yaseen S., Kamal A., Butt B., Matloob H., Alamri S.A.M., Alrumman S.A., Eid E.M., Shahbaz M. (2021). Combined use of endophytic bacteria and pre-sowing treatment of thiamine mitigates the adverse effects of drought stress in wheat (*Triticum aestivum* L.) cultivars. Sustainability.

[4] Bukhari S.A.B.H., Lalarukh I., Amjad S.F., Mansoora N., Naz M., Naeem M., Bukhari S.A., Shahbaz M., Ali S.A., Marfo T.D., Danish S., Datta R., Fahad S. (2021). Drought stress alleviation by potassium-nitrate-containing chitosan/montmorillonite microparticles confers changes in spinacia oleracea L. Sustainability.

[5] Rafeeq H., Arshad M.A., Amjad S.F., Ullah M.H., Imran H.M., Khalid R., Yaseen M., Ajmal H. (2020). Effect of nickel on different physiological parameters of raphanus sativus. Int. J. Sci. Res. Publ..

[6] Yaseen S., Amjad S.F., Mansoora N., Kausar S., Shahid H., Alamri S.A.M., Alrumman S.A., Eid E.M., Ansari M.J., Danish S., Datta R. (2021). Supplemental effects of biochar and foliar application of ascorbic acid on physio-biochemical attributes of barley (hordeum vulgare L.) under cadmium-contaminated soil. Sustainability.

[7] Hanin M., Ebel C., Ngom M., Laplaze L., Masmoudi K. (2016). New insights on plant salt tolerance mechanisms and their potential use for breeding. Front. Plant Sci..

[8] Ayman M., Metwally S., Mancy M., Abd alhafez A. (2020). Beneficial role of bagasse biochar on maize plants grown in sandy soil. J. Product. Dev..

[9] Abd El-Hack M.E., Ashour E.A., Aljahdali N., Zabermawi N.M., Baset S.A., Kamal M., Bassiony S.S. (2024). Does the dietary supplementation of organic nano-zinc as a growth promoter impact broiler's growth, carcass and meat quality traits, blood metabolites and cecal microbiota?. Poult. Sci..

[10] Ayman M., Fawzy Z.F. (2023). Enhancing the availability of potassium in new Egyptian soils using biochar produced from olive stone waste. IOP Conf. Ser. Earth Environ. Sci..

[11] Shokuhifar Y., Ghahsareh A.M., Shahbazi K., Tehrani M.M., Besharati H. (2023). Biochar and wheat straw affecting soil chemistry and microbial biomass carbon countrywide. Biomass Convers. Biorefinery.

[12] Huang S., Huang P., Hareem M., Tahzeeb-ul-Hassan M., Younis U., Dawar K., Fahad S., Salmen S.H., Ansari M.J., Danish S. (2024). Evaluating the hidden potential of deashed biochar in mitigating salinity stress for cultivation of fenugreek. Sci. Rep..

[13] Abd El-Hack M.E., Ashour E.A., Baset S.A., Kamal M., Swelum A.A., Suliman G.M., Bassiony S.S. (2024). Effect of dietary supplementation of organic selenium nanoparticles on growth performance and carcass traits of broiler chickens. Biol. Trace Elem. Res..

[14] Ruiu L. (2020). Plant-growth-promoting bacteria (PGPB) against insects and other agricultural pests. Agronomy.

[15] Stegelmeier A.A., Rose D.M., Joris B.R., Glick B.R. (2022). The use of PGPB to promote plant hydroponic growth. Plants.

[16] Poria V., Dębiec-Andrzejewska K., Fiodor A., Lyzohub M., Ajijah N., Singh S., Pranaw K. (2022). Plant Growth-Promoting Bacteria (PGPB) integrated phytotechnology: a sustainable approach for remediation of marginal lands. Front. Plant Sci..

[17] Ajijah N., Fiodor A., Pandey A.K., Rana A., Pranaw K. (2023). Plant growth-promoting bacteria (PGPB) with biofilm-forming ability: a multifaceted agent for sustainable agriculture. Diversity.

[18] Costa S.F., Martins D., Agacka-Mołdoch M., Czubacka A., de Sousa Araújo S. (2018).

[19] Khan N., Humm E.A., Jayakarunakaran A., Hirsch A.M., Hirsch A.M. (2023). Reviewing and renewing the use of bene fi cial root and soil bacteria for plant growth and sustainability in nutrient-poor , arid soils. Front. Plant Sci..

[20] Mishra P., Bhattacharya A., Verma P., Bharti C., Arora N.K., Arora N.K., Bouizgarne B. (2022). Saline Agroecosystems BT - Microbial BioTechnology for Sustainable Agriculture Volume 1.

[21] Meng D., Yuan M.M., Li J. (2023). Editorial: microbe assisted plant resistance to abiotic stresses. Front. Plant Sci..

[22] Boro M., Sannyasi S., Chettri D., Verma A.K. (2022). Microorganisms in biological control strategies to manage microbial plant pathogens: a review. Arch. Microbiol..

[23] Ali S.S., Kornaros M., Manni A., Al-Tohamy R., El-Shanshoury A.E.-R.R., Matter I.M., Elsamahy T., Sobhy M., Sun J. (2021). Biofertilizers.

[24] Orozco-Mosqueda M. del C., Santoyo G., Glick B.R. (2023). Recent advances in the bacterial phytohormone modulation of plant growth. Plants.

[25] Orozco-Mosqueda M. del C., Glick B.R., Santoyo G. (2020). ACC deaminase in plant growth-promoting bacteria (PGPB): an efficient mechanism to counter salt stress in crops. Microbiol. Res..

[26] Khalid M., Rehman H.M., Ahmed N., Nawaz S., Saleem F., Ahmad S., Uzair M., Rana I.A., Atif R.M., Zaman Q.U., Lam H.-M. (2022). Using exogenous melatonin, glutathione, proline, and Glycine betaine treatments to combat abiotic stresses in crops. Int. J. Mol. Sci..

[27] Hosseinifard M., Stefaniak S., Ghorbani Javid M., Soltani E., Wojtyla Ł., Garnczarska M. (2022). Contribution of exogenous proline to abiotic stresses tolerance in plants: a review. Int. J. Mol. Sci..

[28] Naz M., Hussain S., Ashraf I., Farooq M. (2023). Exogenous application of proline and phosphorus help improving maize performance under salt stress. J. Plant Nutr..

[29] Irshad I., Anwar-Ul-Haq M., Akhtar J., Maqsood M. (2024). Enhancing maize growth and mitigating salinity stress through foliar application of proline and glycine betaine. Pakistan J. Bot..

[30] Abd El-Hack M.E., Kamal M., Altaie H.A., Youssef I.M., Algarni E.H., Almohmadi N.H., Abukhalil M.H., Khafaga A.F., Alqhtani A.H., Swelum A.A. (2023). Peppermint essential oil and its nano-emulsion: potential against aflatoxigenic fungus *Aspergillus flavus* in food and feed. Toxicon.

[31] Prasad S., Tyagi A.K., Aggarwal B.B. (2014). Recent developments in delivery, bioavailability, absorption and metabolism of curcumin: the golden pigment from golden spice. Cancer Res. Treat..

[32] Zheng Y., Jiao C., Sun H., Rosli H.G., Pombo M.A., Zhang P., Banf M., Dai X., Martin G.B., Giovannoni J.J., Zhao P.X., Rhee S.Y., Fei Z. (2016). iTAK: a program for genome-wide prediction and classification of plant transcription factors, transcriptional regulators, and protein kinases. Mol. Plant.

[33] Ayman M., Metwally S., Mancy M., Abd alhafez A. (2020). Influence of nano–silica on wheat plants grown in salt–affected soil. J. Product. Dev..

[34] Ahmed K.B.M., Khan M.M.A., Shabbir A., Ahmad B., Uddin M., Azam A. (2023). Comparative effect of foliar application of silicon, titanium and zinc nanoparticles on the performance of vetiver- a medicinal and aromatic plant. Silicon.

[35] Dawood H., Iqbal M., Azhar M., Ahmad H., Dawood H. (2019). Texture-preserving denoising method for the removal of random-valued impulse noise in gray-scale images. Opt. Eng..

[36] Fahmy M.A., Salem S.H., Qattan S.Y.A., Abourehab M.A.S., Ashkan M.F., Al-Quwaie D.A., Abd El-Fattah H.I., Akl B.A. (2022). Biodegradation of chlorantraniliprole and flubendiamide by some bacterial strains isolated from different polluted sources. Processes.

[37] Atlas R.M., Synder J.W. (2011). Man. Clin. Microbiol..

[38] Ouided B., Abderrahmane B. (2013). Isolation and characterization of glyphosate-degrading bacteria from different soils of Algeria. African J. Microbiol. Res..

[39] John E.M., Sreekumar J., Jisha M.S. (2016). Optimization of chlorpyrifos degradation by assembled bacterial consortium using response surface methodology, soil sediment contam. Int. J..

[40] Miralles D., Slafer G. (1991). A simple model for non-destructive estimates of leaf area in wheat. Cereal Res. Commun..

[41] Coombs J., Hall D.O., Long S.P., Scurlock J.M.O. (1985).

[42] Fageria N.K. (2012).

[43] Estefan G., Sommer R., Ryan J. (2013). https://hdl.handle.net/20.500.11766/7512.

[44] Parkinson J.A., Allen S.E. (1975). A wet oxidation procedure suitable for the determination of nitrogen and mineral nutrients in biological material. Commun. Soil Sci. Plant Anal..

[45] Pandian K., Subramaniayan P., Gnasekaran P., Chitraputhirapillai S. (2016). Effect of biochar amendment on soil physical, chemical and biological properties and groundnut yield in rainfed Alfisol of semi-arid tropics. Arch. Agron Soil Sci..

[46] Gul S., Javed S., Azeem M., Aftab A., Anwaar N., Mehmood T., Zeshan B. (2023). Application of Bacillus subtilis for the alleviation of salinity stress in different cultivars of wheat (tritium aestivum L.). Agronomy.

[4] El-Bassiouny H.M.S., Mahfouze H.A., Abdallah M.M.S., Bakry B.A., El-Enany M.A.M. (2022). Physiological and molecular response of wheat cultivars to titanium dioxide or zinc oxide nanoparticles under water stress conditions. Int. J. Agron..

[48] Abd El-Hack M.E., El-Saadony M.T., Saad A.M., Salem H.M., Ashry N.M., Ghanima M.M.A., Shukry M., Swelum A.A., Taha A.E., El-Tahan A.M., AbuQamar S.F. (2022). Essential oils and their nanoemulsions as green alternatives to antibiotics in poultry nutrition: a comprehensive review. Poult. Sci..

[49] Zafar S., Perveen S., Kamran Khan M., Shaheen M.R., Hussain R., Sarwar N., Rashid S., Nafees M., Farid G., Alamri S., Shah A.A., Javed T., Irfan M., Siddiqui M.H. (2022). Effect of zinc nanoparticles seed priming and foliar application on the growth and physio-biochemical indices of spinach (Spinacia oleracea L.) under salt stress. PLoS One.

[50] Kataria S., Jain M., Rastogi A., Živčák M., Brestic M., Liu S., Tripathi D.K. (2019). Nanomater. Plants, Algae Microorg.

[51] Mustafa N., Raja N.I., Ilyas N., Ikram M., Mashwani Z.-R., Ehsan M. (2021). Foliar applications of plant-based titanium dioxide nanoparticles to improve agronomic and physiological attributes of wheat (*Triticum aestivum* L.) plants under salinity stress. Green Process. Synth..

[52] Li Y., Xi K., Liu X., Han S., Han X., Li G., Yang L., Ma D., Fang Z., Gong S., Yin J., Zhu Y. (2023). Silica nanoparticles promote wheat growth by mediating hormones and sugar metabolism. J. Nanobiotechnology.

[53] Saddiq M.S., Iqbal S., Hafeez M.B., Ibrahim A.M.H., Raza A., Fatima E.M., Baloch H., Jahanzaib, Woodrow P., Ciarmiello L.F. (2021). Effect of salinity stress on physiological changes in winter and spring wheat. Agronomy.

[54] Poustini K., Siosemardeh A. (2004). Ion distribution in wheat cultivars in response to salinity stress. F. Crop. Res..

[55] Saghir S.A., Al-Gabri N.A., Khafaga A.F., El-Shaer N.H., Alhumaidh K.A., Elsadek M.F., Ahmed B.M., Alkhawtani D.M., Abd El-Hack M.E. (2019). Thymoquinone-PLGA-PVA nanoparticles ameliorate bleomycin-induced pulmonary fibrosis in rats via regulation of inflammatory cytokines and iNOS signaling. Animals.

[56] Hassanpouraghdam M.B., Mehrabani L.V., Tzortzakis N. (2020). Foliar application of nano-zinc and iron affects physiological attributes of Rosmarinus officinalis and quietens NaCl salinity depression. J. Soil Sci. Plant Nutr..

[57] Ragab S.M., Turoop L., Runo S., Nyanjom S. (2022). Nanoparticle treatments based on zinc oxide and moringa oleifera leaf extracts alleviate salinity stress in faba bean (Vicia faba L.). J. Agric. Chem. Environ..

[58] Palansooriya K.N., Wong J.T.F., Hashimoto Y., Huang L., Rinklebe J., Chang S.X., Bolan N., Wang H., Ok Y.S. (2019). Response of microbial communities to biochar-amended soils: a critical review. Biochar.

[59] Ajeng A.A., Abdullah R., Ling T.C., Ismail S., Lau B.F., Ong H.C., Chew K.W., Show P.L., Chang J.-S. (2020). Bioformulation of biochar as a potential inoculant carrier for sustainable agriculture. Environ. Technol. Innov..

[60] Singh R.P., Jha P.N. (2016). The multifarious PGPR Serratia marcescens CDP-13 augments induced systemic resistance and enhanced salinity tolerance of wheat (*Triticum aestivum* L.). PLoS One.

[61] Boretti A., Rosa L. (2019). Reassessing the projections of the world water development report. Npj Clean Water.

[62] Tomczyk A., Sokołowska Z., Boguta P. (2020). Biochar physicochemical properties: pyrolysis temperature and feedstock kind effects. Rev. Environ. Sci. Bio/Technology.

[63] Shemawar A. Mahmood, Hussain S., Mahmood F., Iqbal M., Shahid M., Ibrahim M., Ali M.A., Shahzad T. (2021). Toxicity of biogenic zinc oxide nanoparticles to soil organic matter cycling and their interaction with rice-straw derived biochar. Sci. Rep..

[64] Babaei K., Seyed Sharifi R., Pirzad A., Khalilzadeh R. (2017). Effects of bio fertilizer and nano Zn-Fe oxide on physiological traits, antioxidant enzymes activity and yield of wheat (*Triticum aestivum* L.) under salinity stress. J. Plant Interact..

[65] Hafez A., Nassef E., Fahmy M., Elsabagh M., Bakr A., Hegazi E. (2020). Impact of dietary nano-zinc oxide on immune response and antioxidant defense of broiler chickens. Environ. Sci. Poll Res..

[66] Greenway H., Munns R. (1980). Mechanisms of salt tolerance in nonhalophytes. Annu. Rev. Plant Physiol..

[67] Nadeem S.M., Zahir Z.A., Naveed M., Asghar H.N., Arshad M. (2010). Rhizobacteria capable of producing ACC‐deaminase may mitigate salt stress in wheat. Soil Sci. Soc. Am. J..

[68] Chakraborty U., Chakraborty B.N., Chakraborty A.P., Dey P.L. (2013). Water stress amelioration and plant growth promotion in wheat plants by osmotic stress tolerant bacteria. World J. Microbiol. Biotechnol..

[69] Akram M.S., Shahid M., Tariq M., Azeem M., Javed M.T., Saleem S., Riaz S. (2016). Deciphering Staphylococcus sciuri SAT-17 mediated anti-oxidative defense mechanisms and growth modulations in salt stressed maize (Zea mays L.). Front. Microbiol..

[70] Sharma S., Kulkarni J., Jha B. (2016). Halotolerant rhizobacteria promote growth and enhance salinity tolerance in peanut. Front. Microbiol..

[71] Abd_Allah E.F., Hashem A., Alam P., Ahmad P. (2019). Silicon alleviates nickel-induced oxidative stress by regulating antioxidant defense and glyoxalase systems in mustard plants. J. Plant Growth Regul..

[72] Ullah A., Akbar A., Luo Q., Khan A.H., Manghwar H., Shaban M., Yang X. (2019). Microbiome diversity in cotton rhizosphere under normal and drought conditions. Microb. Ecol..

